# Perceived Partner Responsiveness Attenuate the Link Between Menopausal Symptoms and Depressed Affect

**DOI:** 10.1093/geroni/igaa057.985

**Published:** 2020-12-16

**Authors:** Kayeon Lee, Hey Jung Jun, Susanna Joo, Sun Ah Lee

**Affiliations:** Yonsei University, Seoul, Republic of Korea

## Abstract

The years around menopause are the time that associates not only with hormonal changes but with psychological and social transitions, and previous studies have consistently revealed the relationship between menopause and depression. The present study examined the moderating effect of perceived partner responsiveness (PPR) on the association between menopausal symptoms (MS) and depressed affect (DA). The sample was middle-aged climacteric women (N = 754, Age=49-60) from the second wave of Midlife in the United States (MIDUSⅡ). Measurement for MS consisted of the frequency of five symptoms in the past 30 days (insomnia, heavy sweating, painful intercourse, hot flashes, and irritability). PPR was assessed using three items matched the core components of responsiveness (understanding, validating, and caring). Results revealed that there were significant interactions between menopausal symptoms and PPR (b = 0.05, p < 0.039). Specifically, the level of elevation of DA in response to MS was smaller in women with higher levels of PPR (b = 2.93, p < 0.001) than in those with lower levels of PPR (b =3.05, p < 0.001). According to the region of significance analysis, the coefficients of MS on DA were significant within the -2SD to +2SD range of PPR, but it decreased as the PPR increased. Findings suggest that partners’ careful responsiveness may mitigate the detrimental effects of MS on DA among climacteric women.

